# Human and mouse cortical astrocytes: a comparative view from development to morphological and functional characterization

**DOI:** 10.3389/fnana.2023.1130729

**Published:** 2023-04-17

**Authors:** Elisa Degl’Innocenti, Maria Teresa Dell’Anno

**Affiliations:** ^1^Fondazione Pisana per la Scienza ONLUS, San Giuliano Terme, Italy; ^2^Department of Translational Research and New Technologies in Medicine and Surgery, University of Pisa, Pisa, Italy

**Keywords:** astrogenesis, corticogenesis, evolution, astrocyte morphology, astrocyte species-specificity, astrocyte function

## Abstract

The vision of astroglia as a bare scaffold to neuronal circuitry has been largely overturned. Astrocytes exert a neurotrophic function, but also take active part in supporting synaptic transmission and in calibrating blood circulation. Many aspects of their functioning have been unveiled from studies conducted in murine models, however evidence is showing many differences between mouse and human astrocytes starting from their development and encompassing morphological, transcriptomic and physiological variations when they achieve complete maturation. The evolutionary race toward superior cognitive abilities unique to humans has drastically impacted neocortex structure and, together with neuronal circuitry, astrocytes have also been affected with the acquisition of species-specific properties. In this review, we summarize diversities between murine and human astroglia, with a specific focus on neocortex, in a panoramic view that starts with their developmental origin to include all structural and molecular differences that mark the uniqueness of human astrocytes.

## Introduction

The notion of neuroglia as glue embedding different cellular components of the central nervous system (CNS) was first proposed by Virchow (1856, 1858). Subsequently, in the second half of the 19th century, the neuroanatomist Santiago Ramón y Cajal, was able to visualize astrocytes for the first time by using a gold and mercury chloride-sublimate staining (Ramón y Cajal, 1913) labeling a protein later identified by Eng et al. (1971) as glial fibrillary acidic protein (GFAP). These pioneering discoveries paved the path to countless studies that served to highlight the plethora of functions operated by astrocytes in the CNS, such as synapse maturation and elimination (Chung et al., 2015), ion and neurotransmitters homeostasis (Simard and Nedergaard, 2004), regulation of functional hyperemia (Macvicar and Newman, 2015), and modulation of synaptic plasticity (Bains and Oliet, 2007; Ota et al., 2013).

Most of the information currently accessible on astrocytes has been collected from animal models, especially from rodents. While these studies proved invaluable to gain an insight on the multiple functions operated by astrocytes, an increasing body of evidence is pointing out several divergences between astrocytes across species, both at the morphological and at the molecular level (Oberheim et al., 2009; Zhang et al., 2016; Falcone and Martínez-Cerdeño, 2023). In particular, considerable differences have been highlighted between adult human and murine astrocytes.

The cerebral cortex has been object of systematic investigations at this regard, in an attempt to delineate, at a cellular level, the contribution of non-neuronal cells to the cognitive capacities that distinguish humans. Data have shown not only a larger abundance of astrocytes in humans, but also the existence of human-specific astrocyte types endowed with distinctive shapes and diverse functions (Oberheim et al., 2009; Vasile et al., 2017; Falcone and Martínez-Cerdeño, 2023). The contemporary evo-devo approach uses developmental principles to obtain a glimpse into how human neocortex may have evolved (Rakic, 2009) and on this basis, it appears conceivable that also inter-specific differences in astrocytes may origin during development.

In this context, the purpose of this review is to trace an outline of the currently available notions on the differences between human and mouse astrocytes development, with a specific focus on cerebral cortex, and eventually, extending the comparison to mature cells on a morphological, molecular, and functional perspective.

## Astroglia development in mouse and human cerebral cortex

**FIGURE 1:**
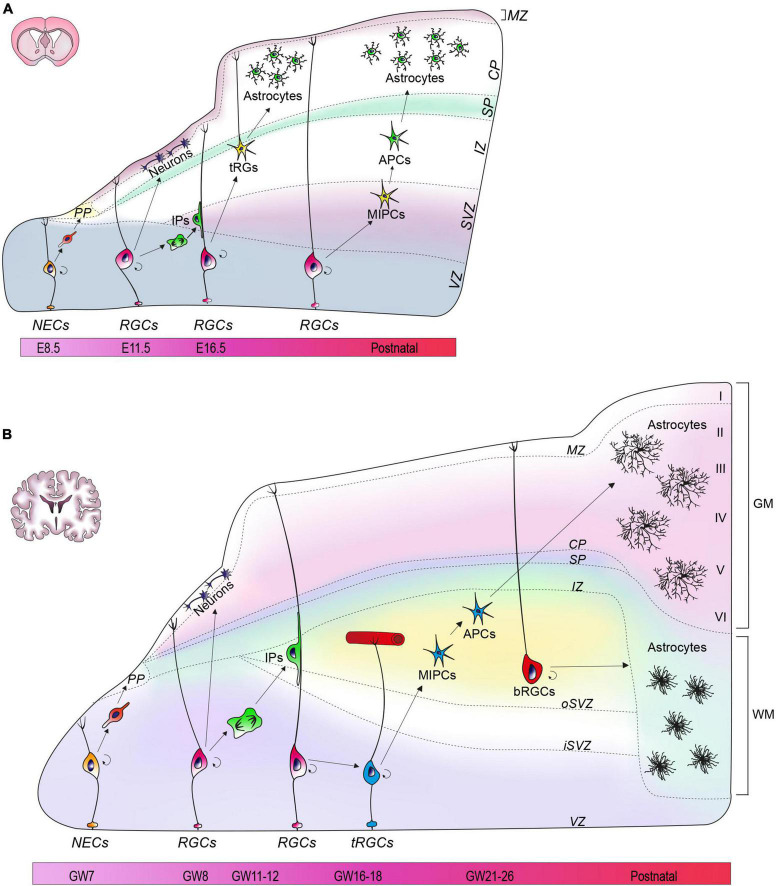
Astrogenesis in mouse and human cortex. **(A)** At E8.5, neuroepithelial cells (NECs) are located in the ventricular zone (VZ). Around E10.5, the preplate (PP) appears and later divides into a marginal zone (MZ), a subplate (SP), and cortical plate (CP). Radial glial cells (RGCs) begin to generate astrocytes at E18.5. During the first wave of astrogenesis, RGCs give rise to transforming RGCs (tRG) that reside in the intermediate zone (IZ). In the second wave, intermediate progenitors (MIPCs) residing in the subventricular zone (SVZ) generate astrocytic progenitors (APCs). **(B)** At GW7, a single layer of NECs characterizes the developing human neural tube. Around GW7-8 the PP appears, and then divides into MZ, CP and SP. Astrogenesis begins around GW21-26 from two different sources: truncated RGCs (tRGCs) and basal RGCs (bRGCs). The tRGCs maintain the ventricular process and contact vasculature in the oSVZ, while detaching from the pial surface. The tRGCs generate multipotent intermediate progenitors (MIPCs) that localize between the oSVZ and the inner SVZ (iSVZ). From the MIPCs, APCs arise and eventually differentiate into gray matter (GM) astrocytes. bRGCs detach their basal process from the VZ and reside in the outer SVZ (oSVZ), where they generate white matter (WM) astrocytes.

The neural tube is the primordial structure of the CNS and, in the earliest developmental stages, is composed by a single layer of neuroepithelial cells (NECs). These cells constitute the ventricular zone (VZ) of the neural tube and are the founders from which all neurons and glial cells of the adult CNS will be generated. Until the seventh post-conceptional week in humans, or day 8 of embryonic development in mice (E8), NECs undergo primarily symmetric divisions in order to expand the stem cell pool (Baggiani et al., 2020). Subsequently, a small fraction of NECs undergoes asymmetric divisions to generate the first wave of post-mitotic neurons that migrate radially into a transient structure called preplate (PP) (Figure 1A) (Gao et al., 2013). As development proceeds, NECs transform into radial glia cells (RGCs) which exhibit a typical bipolar morphology with an apical process touching the ventricular edge, and a long process extending toward the pia (Figure 1A) (Miyata et al., 2001; Noctor et al., 2004). RGCs divide, but unlike NECs, the divisions of RGCs are mostly asymmetric, giving rise to a daughter RGC, an intermediate progenitor (IP) cell, or a nascent neuron that will subsequently migrate toward the pial surface. IPs originated from RGCs then delaminate from the VZ and migrate to reach the subventricular zone (SVZ) where they undergo additional cycles of symmetric divisions to generate neurons (Figure 1A). In humans, IPs undergo numerous rounds of division before starting neuronal differentiation, whereas in mouse divisions are limited to one cycle (Hansen et al., 2010; Lamonica et al., 2013). Exploiting the processes of RGCs as a guide for radial migration, newborn neurons split the PP region into three areas: a more superficial marginal zone (MZ), that will eventually become the future layer 1 of the cortex, an intermediate area called cortical plate (CP), and a deeper and transient subplate (SP) (Figure 1A). As a result of successive waves of migration, newly generated neurons migrate past the existing born neurons and occupy more superficial layers in the CP, thus generating layers 2–6 of the forming cortex, according to an inside-out pattern that characterizes cortical lamination (Rakic, 1988; Bronner and Hatten, 2013; Mukhtar and Taylor, 2018).

In mice, at E16, RGCs lose their neurogenic potential in favor of a progressive gliogenic capacity that reaches its peak at the postnatal day (P) 6 and starts decreasing at P28 (Ge et al., 2012). Astrocytes in mouse originate from two sites: the VZ and the SVZ. In the VZ, the RGCs translocate, detaching their process from the ventricular surface of the cortex, and lifting their soma toward the pial surface, thus acquiring the novel identity of transforming RG (tRG) (Figure 1A) (Nadarajah et al., 2001; Noctor et al., 2004; Deneen et al., 2020). Eventually, tRG terminally differentiate and give rise to protoplasmic and fibrous astrocytes (Figure 1A) (Deneen et al., 2020). The second wave of astrogenesis occurs in SVZ during the postnatal period, leading to the generation of gray matter (GM) astrocytes (Gressens et al., 1992; Tabata, 2015). In this area, RGCs generate multipotent intermediate progenitors (MIPCs) molecularly distinguishable for the expression of ASCL1, EGFR, OLIG2, and MKI67 (Li X. et al., 2021). Subsequently, MIPCs originate both astrocyte progenitors (APCs) (Figure 1A) and oligodendrocyte progenitors (OPCs), which terminally differentiate into mature cells (Li X. et al., 2021). A subset of oligodendrocyte precursors, named NG2 cells, can also generate protoplasmic astrocytes prenatally (∼ E17.5) before the acquisition of a fully differentiated phenotype (Nishiyama et al., 2016; Guo et al., 2021). Once precursors are specified, the last step is migration toward their final location. Precursors of the VZ migrate through the direct transformation into tRG with the consequent retraction of radial fibers that pushes the soma upward (Tabata, 2015), whereas SVZ precursors migrate radially into both white matter (WM) and GM (Jacobsen and Miller, 2003). When the progenitors are positioned in their final location, they undergo numerous rounds of proliferation, especially during the first three postnatal weeks, before entering terminal differentiation (Ge et al., 2012; Molofsky and Deneen, 2015; Deneen et al., 2020).

Human astrogenesis appears to occur mostly in the second half of gestation, with a variability of a few weeks correlated to the anatomical area (Choi and Lapham, 1978; Holst et al., 2019), and seems to persist in the postnatal period (Roessmann and Gambetti, 1986). In humans, as well as in mice, the principal neural stem cell niches for astrocytes are in the VZ and SVZ. In human VZ, around gestational week (GW) 16–18, a subset of RGCs generate the so-called truncated RGCs (tRGCs), which are characterized by the loss of contact with the pial surface, and by the abrupt termination of basal processes on the blood vessels of the oSVZ (Figure 1B) (DeAzevedo et al., 2003; Nowakowski et al., 2016; Holst et al., 2019). tRGCs give rise to MIPCs residing in the inner fibers layer, which are characterized by the expression of EGFR, thought to mediate the initiation of gliogenesis, ASCL1, OLIG1, and OLIG2 (Yang et al., 2022). Similar to mouse astrogenesis, human MIPCs will subsequently give rise to APCs and OPCs that will terminally differentiate into astrocytes and oligodendrocytes, respectively (Figure 1B) (Yang et al., 2022).

In humans, the SVZ further subdivides into outer SVZ (oSVZ), and inner SVZ (iSVZ) by means of an internal layer of fibers (Figure 1B) (Rakic, 1988; Bystron et al., 2008; Bronner and Hatten, 2013; Mukhtar and Taylor, 2018; Molnár et al., 2019). This subdivision and the vast amplification of the oSVZ are two prominent elements of difference compared to developing mouse cortex and are also acknowledged as responsible of the gyrification that distinguishes human from mouse brains (Namba and Huttner, 2017). In the human oSVZ, a peculiar type of RGCs not described in mouse, are named outer or basal RGCs (bRGCs). bRGCs are characterized by the loss of their connections with the ventricular surface and by retention of basal processes facing the pia (Figure 1B) (Ortega et al., 2018). Observations conducted in non-human primates have outlined that, after completion of neurogenesis, bRGCs acquire a prominent gliogenic capacity which has an important role for the ‘fanning out’ of the cortex, the enlargement of the cerebrum and development of convolutions (Rash et al., 2019).

By means of human oraganotypic brain slices collected at the onset of astrogliogenesis (GW18-23), a recent work has showed that astrocytes originated from tRGCs (VZ), and bRGCs (oSVZ) follow distinct fates in the human cortex, thus pointing out for the first time, the identification of two separate niches (Allen et al., 2022). Specifically, astrocytes originated in the VZ typically endowed with dense and bulbous processes are eventually localized in the CP (∼70%) and in the SP (∼29%), prevalently giving rise to GM astrocytes. Astrocytes generated in the oSVZ, on the other hand, have smooth processes and for the vast majority remain in the SVZ, where they generate WM astrocytes. These two populations have also been molecularly defined by mean of RNA sequencing which identified in *ITGB4* and *ANGPTL4* the candidate markers of VZ- and oSVZ-originated astrocytes, respectively (Figure 1B) (Allen et al., 2022).

## Cortical astrocytes: mouse and human morphometric assessment

The evolution of the CNS has driven an increase in brain size (DeFelipe, 2011). Like neurons, astrocytes changed in shape, size and number becoming progressively more specialized in evolved species (Falcone and Martínez-Cerdeño, 2023). Accordingly, the astrocyte to neuron ratio has also evolved from 1:3 in the mouse cortex to 1:1.4 in the human cortex, an increase arguably ascribed to the presence of more sophisticated neuronal networks (Bass et al., 1971; Nedergaard et al., 2003). Disparity from a numerical point of view is also accompanied by morphological divergence (Oberheim et al., 2009; Vasile et al., 2017). Human astrocytes are much larger, more complex, and more heterogeneous that their murine counterpart, with species-specific subtypes that have been only found in primates (Oberheim et al., 2009; Vasile et al., 2017; Falcone and Martínez-Cerdeño, 2023). Of note, four types of astrocytes have been observed in humans: interlaminar, varicose projections, protoplasmic and fibrous. With the exception of varicose projections astrocytes, all the other types were also found in the mouse brain, with several morphometric differences outlined in the section below.

### Interlaminar astrocytes

**FIGURE 2:**
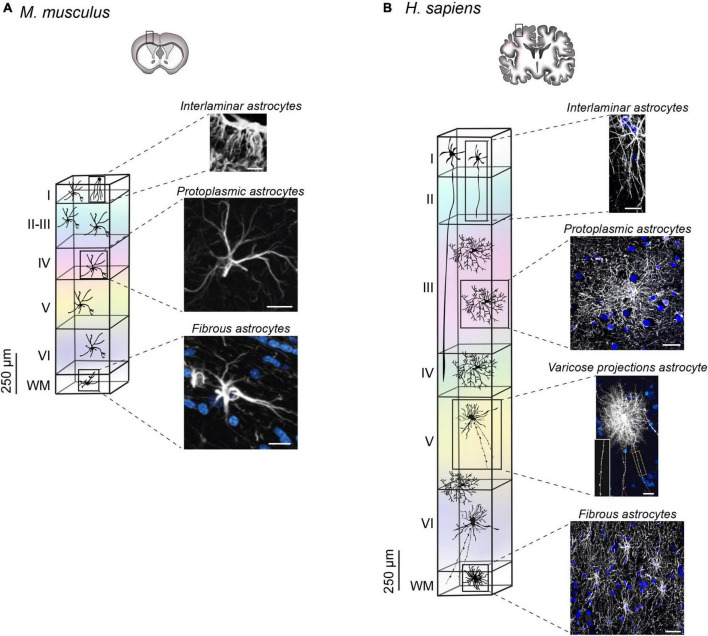
Arrangement and morphology of human and mouse astrocytes. **(A)** In mice, three types of astrocytes have been identified: protoplasmic, fibrous and rudimental interlaminar. Interlaminar astrocytes in mouse cortex reside in layer 1 with very short processes identified by immunohistochemical labeling of GFAP [image adapted and modified from Falcone et al. (2019); scale bar 50 μm]. Protoplasmic astrocytes localize in all layers, whereas fibrous astrocytes are found in WM. Both subtypes are labeled by immunofluorescence staining of GFAP image adapted and modified from Oberheim et al. (2009). Scale bar for protoplasmic astrocyte 20 μm, scale bar for fibrous astrocyte 10 μm. **(B)** Human astrocyte spatial arrangement in the cortex depends on the cellular subtype. The soma of interlaminar astrocytes localizes in layer 1 with projections reaching layers 2–4 (scale bar 15 μm). Protoplasmic astrocytes reside in layers 2–6 (scale bar 30 μm), whereas varicose projection astrocytes are found in layers 5–6 and in WM (scale bar 20 μm). Fibrous astrocytes localize in the WM (scale bar 30 μm). Astrocyte images are adapted from Degl’Innocenti et al. (2022) (immunofluorescence staining of GFAP) with the exception of varicose projection astrocyte image adapted and modified from Oberheim et al. (2009), generated by diolistic labeling. All images were readapted with permission (Copyright 2009 Society for Neuroscience).

Originally identified by Andriezen (1893) and Retzius (1894), interlaminar astrocytes reside in layer 1 of the cerebral cortex, and exhibit long and tortuous varicosity-free processes that typically terminate in layers 2–4 (Colombo and Reisin, 2004). Recently, two types of interlaminar astrocytes have been described: pial and subpial. Pial interlaminar astrocytes are in direct contact with the pial surface and present an inverted pyramidal shaped soma. Conversely, subpial astrocytes, with their rounded soma, are located in the upper layer 1 with processes contacting the pia. Both types were detected in human and mouse cortex, although in the mouse they present a very rudimental morphology with processes limited to layer 1 (Figure 2A) (Falcone et al., 2019). The degree of complexity of human interlaminar astrocytes has been pointed out regarding both the total number of processes (27.9 in *H. sapiens versus* 5.8 in *M. musculus*), and their overall length that reaches 593.4 μm in human, while being only 133.4 μm in mouse (Falcone et al., 2019).

### Varicose projection astrocytes

Varicose projection astrocytes are detectable only in humans and other apes in layers 5–6 and in the WM of the cortex (Falcone et al., 2021). They are characterized by the presence of short spiny processes and 1–5 mm long projections with prominent, evenly spaced varicosities. The long processes, span in all directions and often contact vessels (Figure 2B) (Oberheim et al., 2009). Their processes are less tortuous respect to the interlaminar astrocytes and are less branched in comparison with the protoplasmic astrocytes, thus arguably establishing a lower number of synaptic contacts.

### Protoplasmic astrocytes

Protoplasmic astrocytes reside in layers 2–6 of human and mouse cortex and are the most common astrocyte type in the GM. In humans they exhibit a largely more complex arborization with approximately 37.5 processes, which can measure up to 100 μm (Figure 2B). The number of branches goes down to 3.75 per cell in mice, with an approximate length of 39 μm (Figure 2A) (Oberheim et al., 2009). Because of their finely articulated branching, human protoplasmic astrocytes cover a high number of synapses (270,000 to 2 million), thus facilitating the modulation of inter-neuronal communication and local information integration. Each protoplasmic astrocyte retains its own anatomical space. However, in humans, the anatomical borders are less preserved, compared to their rodent counterpart and present an area of overlap of about ∼205 μm^2^, which is limited to ∼12 μm^2^ in mice (Oberheim et al., 2009).

### Fibrous astrocytes

Fibrous astrocytes reside in the WM both in mice and humans. They are organized parallel to the axon fibers, on which their perinodal processes terminate by interdigitating in the Ranvier’s nodes (Verkhratsky et al., 2021). From a morphological point of view, they present lobate and oblong nuclei, their unbranched processes are long and very thin (Verkhratsky et al., 2021). The projections generally contact neighboring fibrous astrocytes with overlapping anatomical domains (Oberheim et al., 2009). In humans these astrocytes appear noticeably larger, about two times than in mice (183.2 ± 6.1 μm *versus* 85.6 ± 2.7 μm) (Figures 2A, B). Their function is arguably structural, for the support of the axonal tracts (Oberheim et al., 2009).

## Molecular and functional characterization of human and mouse astrocytes

In recent decades, growing evidence has highlighted the plethora of functions operated by astrocytes. As structural components of the neurovascular unit, astrocytes are essential for the formation and maintenance of the blood brain barrier (BBB) (Virchow, 1858; Bélanger et al., 2011; Cabezas et al., 2014; Macvicar and Newman, 2015), for the transport of cerebrospinal fluid (CSF) in the glymphatic system (Iliff and Nedergaard, 2013; Jessen et al., 2015), and for metabolic support (Bélanger et al., 2011). Astrocytes also notably assist synapse formation and maintenance (Chung et al., 2015), participate in the tripartite synapse (Araque et al., 1999; Perea et al., 2009; Farhy-Tselnicker and Allen, 2018), modulate synaptic plasticity (Bains and Oliet, 2007; Ota et al., 2013), and regulate neurotransmitters uptake and recycling (Sonnewald et al., 1997; Simard and Nedergaard, 2004). For a systematic and general description of all the functions operated by astrocytes we refer to other reviews (Khakh and Sofroniew, 2015; Verkhratsky and Nedergaard, 2018). In the following subsections, we focus primarily on those elements that have been explored in both species and for which differences have been outlined.

### Morphofunctional differences

The morphological heterogeneity pointed out in astrocytes is paralleled by functional diversification. Compared to mouse, protoplasmic astrocytes in the human cortex exhibit a larger and more complex branching of the processes (Oberheim et al., 2009; Han et al., 2013; Vasile et al., 2017). Although the area covered by their projections is extended for an optimal integration of a larger number of synapses, and with a considerable degree of overlap in relation to what is observed in mice, it is confined to a single cortical layer. Intra-layer communication, on the other hand, relies on interlaminar astrocytes, which represent an additional important element of diversity between human and mouse astroglia (Falcone et al., 2019). Initially thought to be a specific subtype of primate brains, interlaminar astrocytes have only been recently added to the list of astrocytes subtypes that can be equally found in mice and humans. Nevertheless, mouse interlaminar astrocytes exhibit very short projections limited to the first cortical layer (Falcone et al., 2019). Conversely, with columnar connections spanning up to 4 cortical layers, the human counterpart covers very distant territories (Falcone et al., 2019). The radial morphology of interlaminar astrocytes may be involved in maintaining the columnar organization and function in the cortex (Colombo and Reisin, 2004). Consequently, species with a more organized columnar structure present a greater number of longer interlaminar processes, as in the case with primates and humans, specifically (Falcone et al., 2019). The range of cellular interactions established by processes is determinant in outlining specific functions. Interlaminar astrocytes have been shown to directly contact pia and capillaries, suggesting a role in the BBB and in facilitating the communication of other cell types with meninges and CSF (Falcone et al., 2019). A peculiar feature of human protoplasmic astrocyte projections that distinguishes them from interlaminar, refers to contacts with nodes of Ranvier, suggestive of a specific participation of this cell type in ionic buffering of extracellular space around the node (Sosunov et al., 2014).

Varicose projection astrocytes have only been found in humans and apes and are a definite element of difference with many other species. Intriguingly, it has been recently observed that they are not a constant element of human astroglia and that they can be more frequently observed in conjunction with varicosities on interlaminar astrocytes (Falcone et al., 2021). On this basis, it has been hypothesized that varicosities on interlaminar astrocytes may be a feature acquired under specific conditions, i.e. stress, aging or disease occurrence and that similar factors may also trigger the appearance of varicose projection astrocytes. Given the similarities with fibrous astrocytes in terms of location and morphological aspect, varicose projection astrocytes may be a modified version of fibrous astrocytes generated in response to specific cues, more likely traumatic injuries, which already proved to provoke varicosities protrusion in astrocytes (Falcone et al., 2021).

### Ion and metabolite homeostasis

A key element for the surveillance of proper synapse transmission is the control of ion and metabolite homeostasis (Verkhratsky and Nedergaard, 2018), a function operated through the formation of astrocytes syncytia (Dermietzel et al., 1989; Rouach et al., 2002; Giaume et al., 2010). Initially thought to be a feature of protoplasmic astrocytes of the GM, astrocytes have demonstrated to establish syncytia also in the WM of the *corpus callosum*, predominantly populated by the fibrous type (Kiyoshi et al., 2018). Gap junctions mediate the connection between neighboring astrocytes within syncytia, an element that is not only structural but also serves to guarantee the spatial redistribution of K^+^ and Na^+^ ions, as well as of nutrients, metabolites, and signaling molecules for the coordination of neuronal activity and brain energy metabolism (Ma et al., 2016). In the mouse, gap junction coupling by means of connexins 30, 43 and 26 appears to be pivotal for the establishment of syncytia (Dermietzel et al., 1989; Charvériat et al., 2021), which have been shown to acquire maturation only postnatally (P15) (Zhong et al., 2023). Evidence suggests that human astrocytes also exhibit gap junction coupling (Bedner et al., 2015), as well as connexin 43 (Aronica et al., 2001), and connexin 30 (Nagy et al., 1999). In the control of ion homeostasis, the cerebral water content regulated by the AQP4 channel is also important. AQP4 is localized on astrocyte endfeet where it mediates diverse functions such as K^+^ buffering, CSF circulation and waste clearance (Nagelhus and Ottersen, 2013). AQP4 exhibits a different degree of polarization in mouse or human astrocytes: in the latter, it appears more densely distributed on the astrocytic membrane, but with a minor degree of polarization on endfeet than in murine cells (Eidsvaag et al., 2017).

Functional differences between human and murine astrocytes have also been delineated from a metabolic point of view. RNA sequencing analyses has pointed out the highest in expression in human astrocytes of *APOC2*, involved in fatty acid metabolism well as of *AMY2B* and *AADAT*, participating in glycogen metabolism and transaminase-mediated excitatory transmission, respectively (Zhang et al., 2016). Mouse astrocytes show a higher expression of genes implicated in mitochondrial respiration, while human astrocytes exhibit increased expression of genes associated with defense response and genes linked to extracellular space and secreted factors (Li J. et al., 2021). These divergences have been associated with a greater resilience of mouse astrocytes to respond to oxidative stress and to the greater susceptibility of human cells to neurodegenerative disorders or acute traumas in which oxidative insult is critical for the pathological process (Li J. et al., 2021).

### Calcium signaling

From a molecular perspective, human astrocytes contain many conserved genes similarly expressed in mouse (Falcone et al., 2021). On the other hand, about 600 genes have been found to be specifically upregulated in human cells and, among them, genes involved in calcium signaling were highly enriched (Zhang et al., 2016). Calcium in astrocytes fulfills a wide number of functions including the release of gliotransmitters for the modulation of the synaptic activity, and the control of vessel diameter in functional hyperemia, therefore it is conceivable that diverse dynamics in intracellular calcium signaling may reflect species-specific needs in terms of synaptic integration (Parpura et al., 1994; Agulhon et al., 2008; Bélanger et al., 2011). For example, the calcium permeable ion channel ryanodine receptor type 3 (RYR3) is strongly enriched in human astrocytes and amplifies the fast propagation of calcium currents by acting on calcium release from endoplasmic reticulum. Similarly, the MRVI1 protein, binding the IP3 receptors, regulates intracellular calcium stores and is also overexpressed in humans (Zhang et al., 2016). These findings are consistent with the reported differences in calcium waves propagation between the two species as observed by Oberheim and coworkers, who reported a significantly slower speed in rodents than in their human counterparts (8.6 ± 0.6 μm/s versus 43.4 ± 4.7 μm/s) (Oberheim et al., 2009). In addition, the responsiveness of astrocytes to ATP and glutamate is also different, with human astrocytes calcium wave transmission being fourfold faster than in mouse (Oberheim et al., 2009). In line with these findings a new astrocytic marker peculiar to humans, and almost completely absent in mouse, has been identified in Centrin-2 (CETN2), a calcium-binding protein with two EF-hand domains and structurally similar to S100B (Degl’Innocenti et al., 2022).

The species-specific selectivity of several proteins involved in calcium intracellular signaling may suggest and evolution-based specification of calcium propagation that may better serve the computational capacities of species, like humans, endowed with superior cognitive abilities. This assumption seems to find confirmation in the work conducted by Han et al. (2013) who performed the engraftment of human glia progenitors in a recipient mouse. With this experiment they demonstrated, not only that the transplanted cells were gap junction-coupled with recipient host cells, but also that the engrafted human astrocytes retained their human characteristics. In particular, transplanted human astrocytes preserved their original morphology, and performed stronger calcium wave propagation, as occurs physiologically in humans; as a result, the chimera animals showed enhanced long-term potentiation with increased cognitive abilities (Han et al., 2013).

## Discussion

The prominent role of astrocytes in outlining intra-species differences between humans and mice can be inferred by studying cerebral cortex. In this area, human astrocytes achieve a level of complexity that drastically differentiates them from mouse astroglia. The larger shape and the wider network of cellular interactions (Oberheim et al., 2009; Vasile et al., 2017; Falcone et al., 2019, 2021), the faster propagation of second messengers (Oberheim et al., 2009), as well as the increased cognitive abilities of mice engrafted with human astrocytes (Han et al., 2013), are all evidences supporting the idea that evolution has pushed toward an enhanced ability of human astrocytes to respond to stimuli and communicate with other cells. However, at present, a limited number of studies have analyzed human fetal brain tissue (Allen et al., 2022), and many questions regarding the developmental processes through which human astrocytes gain their peculiar features remain to be addressed.

It is currently accepted that gliogenesis follows an initial phase of neurogenesis after the so-called “gliogenic switch.” However, unlike rodents (Noctor et al., 2004), neurogenesis and gliogenesis overlap extensively in humans (Malik et al., 2013), and the question of whether a single progenitor within this time window can generate both neurons and glial cells has not been addressed yet. Additionally, the developmental origin of varicose projection astrocytes, peculiar to humans, or the molecular players that sustain the massive elongation of the processes of human interlaminar astrocytes, which instead appear short and rudimental in mouse, still remain to be elucidated.

Calcium signaling is also an important element of difference. Transcriptomic analysis (Zhang et al., 2016), physiology studies (Oberheim et al., 2009), as well as histological characterization of human and mouse brains (Degl’Innocenti et al., 2022), have pointed out the overexpression of species-specific elements correlated to calcium-operated signal transduction. Calcium signals propagate in the astrocytic syncytia through gap junctions (Verkhratsky and Kettenmann, 1996), whose functionality has been assessed in rodent models (Zhong et al., 2023). At present, no correspondent investigations have been conducted in human samples and the electrophysiological properties of human astrocytic syncytia remain an open question.

At this regard, the possibility to generate astrocytes from human stem cells appears as an invaluable tool to investigate intracellular dynamics. Advances in human induced pluripotent stem cell (hIPSC) technology are being adapted to astrocyte research. At present, astrocytes can be generated in 2D layers or even in 3D systems, such as spheroids or organoids (Sloan et al., 2017; Tchieu et al., 2019; Boder and Banerjee, 2021), or can be grown in mouse chimeric brains (Han et al., 2013; Osipovitch et al., 2019). Interestingly, astrocytes generated from patient derived-hiPSC have been shown to recapitulate some pathological phenotypes, such as lipid metabolism dysregulation, altered signaling, or variation in the content of extracellular vesicles (Lin et al., 2018; di Domenico et al., 2019; Varcianna et al., 2019). However, further experimentation is needed to assess the capacity of *in vitro*-cultured astrocytes to recapitulate the molecular heterogeneity of their *in vivo* counterparts, in relation to their regional identity, degree of maturation and susceptibility to oxidative stress (Li J. et al., 2021) as pivotal elements of human astrocyte uniqueness.

Astrocytes are also known components of the neurovascular unit (NVU) (Iadecola, 2017), a fundamental element of the glymphatic system that cannot be investigated in non-vascularized 3D brain organoids. In this regard, recent efforts have been devoted toward the generation of reliable *in vitro* systems recapitulating the NVU structure either incorporating human primary astrocytes with human endothelial and pericyte cells in spheroid systems (Bergmann et al., 2018), or by taking advantage of the microfluidic technology (Maoz et al., 2018; Vatine et al., 2019). Although these approaches allow the study many aspects of the BBB permeability, they lack of anatomical fidelity as the component cells are arranged in spatial organizations that differ from the human brain cytoarchitecture.

The study of freshly resected human brain tissues would offer the possibility to study astrocytes in their original milieu preserving intact cellular connections. Evidence has shown that this system allows the study of astrocytic calcium currents (Oberheim et al., 2009) and implemented protocols have been put in place for the maintenance of human brain sections to help the long-term preservation of intact electrophysiological properties (Schwarz et al., 2017). However, the adaptation of acute brain slices for the study of human astrocytes needs to take into account that the manipulation of the tissue at the time of the operation triggers an injury response that may evoke a reactive state in glial cells (Qi et al., 2019).

Future studies should be devoted to a deep comprehension of the molecular factors that orchestrate human astrogenesis. The knowledge of transcription factors operating in discrete cell niches, or of the gliotrophic molecules that drive the vast morphological, molecular, and functional heterogeneity of these cells is essential for the implementation of *in vitro* systems, enabling the generation of *bona fide* human astrocytes. An always increasing number of evidence is underlining the pivotal role of non-neuronal cells in the neurological diseases (Siracusa et al., 2019), and the poor translability of studies conducted on murine models highlights the urgent need for a reliable tool to identify human astrocyte-specific targets and conceive novel disease-modifying therapies.

## Author contributions

ED wrote the draft. Both authors edited and finalized the manuscript, designed the figures, and approved the submitted version.

## References

[B1] Agulhon C. Petravicz J. McMullen A. B. Sweger E. J. Minton S. K. Taves S. R. (2008). What is the role of astrocyte calcium in neurophysiology? *Neuron* 59 932–946. 10.1016/j.neuron.2008.09.004 18817732 PMC3623689

[B2] Allen D. E. Donohue K. C. Cadwell C. R. Shin D. Keefe M. G. Sohal V. S. (2022). Fate mapping of neural stem cell niches reveals distinct origins of human cortical astrocytes. *Science* 376 1441–1446. 10.1126/science.abm5224 35587512 PMC9233096

[B3] Andriezen W. L. (1893). The neuroglia elements in the human brain. *Br. Med. J.* 2 227–230. 10.1136/bmj.2.1700.227 20754383 PMC2422013

[B4] Araque A. Parpura V. Sanzgiri R. P. Haydon P. G. (1999). Tripartite synapses: Glia, the unacknowledged partner. *Trends Neurosci.* 22 208–215. 10.1016/s0166-2236(98)01349-6 10322493

[B5] Aronica E. Gorter J. A. Jansen G. H. Leenstra S. Yankaya B. Troost D. (2001). Expression of connexin 43 and connexin 32 gap-junction proteins in epilepsy-associated brain tumors and in the perilesional epileptic cortex. *Acta Neuropathol.* 101 449–459. 10.1007/s004010000305 11484816

[B6] Baggiani M. Dell’Anno M. T. Pistello M. Conti L. Onorati M. (2020). Human neural stem cell systems to explore pathogen-related neurodevelopmental and neurodegenerative disorders. *Cells* 9 1–31. 10.3390/cells9081893 32806773 PMC7464299

[B7] Bains J. S. Oliet S. H. (2007). Glia: They make your memories stick! *Trends Neurosci.* 30 417–424. 10.1016/j.tins.2007.06.007 17631972

[B8] Bass N. H. Hess H. H. Pope A. Thalheimer C. (1971). Quantitative cytoarchitectonic distribution of neurons, glia, and DNA in rat cerebral cortex. *J. Comp.Neurol.* 143 481–490. 10.1002/cne.901430405 4945394

[B9] Bedner P. Dupper A. Hüttmann K. Müller J. Herde M. K. Dublin P. (2015). Astrocyte uncoupling as a cause of human temporal lobe epilepsy. *Brain* 138 1208–1222. 10.1093/brain/awv067 25765328 PMC5963418

[B10] Bélanger M. Allaman I. Magistretti P. J. (2011). Brain energy metabolism: Focus on Astrocyte-neuron metabolic cooperation. *Cell Metab.* 14 724–738. 10.1016/j.cmet.2011.08.016 22152301

[B11] Bergmann S. Lawler S. E. Qu Y. Fadzen C. M. Wolfe J. M. Regan M. S. (2018). Blood–brain-barrier organoids for investigating the permeability of CNS therapeutics. *Nat. Protoc.* 13 2827–2843. 10.1038/s41596-018-0066-x 30382243 PMC6673652

[B12] Boder E. J. Banerjee I. A. (2021). Alzheimer’s disease: Current perspectives and advances in physiological modeling. *Bioengineering* 8:211. 10.3390/bioengineering8120211 34940364 PMC8698996

[B13] Bronner M. Hatten M. E. (2013). “Neurogenesis and migration,” in *Fundamental Neuroscience*, 4th Edn, eds Squire L. R. Berg D. Bloom F. Lac S. Ghosh A. Spitzer N. C. (Waltham, MA: Elsevier Inc), 339–361. 10.1016/B978-0-12-385870-2.00015-9

[B14] Bystron I. Blakemore C. Rakic P. (2008). Development of the human cerebral cortex: Boulder committee revisited. *Nat. Rev. Neurosci.* 9 110–122. 10.1038/nrn2252 18209730

[B15] Cabezas R. Ávila M. Gonzalez J. El-Bachá R. S. Báez E. García-Segura L. M. (2014). Astrocytic modulation of blood brain barrier: Perspectives on Parkinson’s disease. *Front. Cell Neurosci.* 8:211. 10.3389/fncel.2014.00211 25136294 PMC4120694

[B16] Charvériat M. Mouthon F. Rein W. Verkhratsky A. (2021). Connexins as therapeutic targets in neurological and neuropsychiatric disorders. *Biochim. Biophys. Acta Mol. Basis Dis.* 1867:166098. 10.1016/j.bbadis.2021.166098 33545299

[B17] Choi B. H. Lapham L. W. (1978). Radial glia in the human fetal cerebrum: A combined Golgi, immunofluorescent and electron microscopic study. *Brain Res.* 148 295–311. 10.1016/0006-8993(78)90721-7 77708

[B18] Chung W. S. Allen N. J. Eroglu C. (2015). Astrocytes control synapse formation, function, and elimination. *Cold Spring Harb. Perspect. Biol.* 7:a020370. 10.1101/cshperspect.a020370 25663667 PMC4527946

[B19] Colombo J. A. Reisin H. D. (2004). Interlaminar astroglia of the cerebral cortex: A marker of the primate brain. *Brain Res.* 1006 126–131. 10.1016/j.brainres.2004.02.003 15047031

[B20] DeAzevedo L. C. Fallet C. Moura-Neto V. Daumas-Duport C. Hedin-Pereira C. Lent R. (2003). Cortical radial glial cells in human fetuses: Depth-correlated transformation into astrocytes. *J. Neurobiol.* 55 288–298. 10.1002/neu.10205 12717699

[B21] DeFelipe J. (2011). The evolution of the brain, the human nature of cortical circuits, and intellectual creativity. *Front. Neuroanat.* 5:29. 10.3389/fnana.2011.00029 21647212 PMC3098448

[B22] Degl’Innocenti E. Poloni T. E. Medici V. Recupero L. Dell’Amico C. Vannini E. (2022). Centrin 2: A novel marker of mature and neoplastic human astrocytes. *Front. Cell Neurosci.* 16:858347. 10.3389/fncel.2022.858347 35573835 PMC9100563

[B23] Deneen B. Akdemir E. S. Huang A. Y. S. (2020). Astrocytogenesis: Where, when, and how. *F1000Res* 9:F1000FacultyRev–233. 10.12688/f1000research.22405.1 32269761 PMC7122459

[B24] Dermietzel R. Traub O. Hwang T. K. Beyer E. Bennett M. V. Spray D. C. (1989). Differential expression of three gap junction proteins in developing and mature brain tissues. *Proc. Natl. Acad. Sci. U.S.A.* 86 10148–10152. 10.1073/pnas.86.24.10148 2557621 PMC298664

[B25] di Domenico A. Carola G. Calatayud C. Pons-Espinal M. Muñoz J. P. Richaud-Patin Y. (2019). Patient-specific iPSC-derived astrocytes contribute to non-cell-autonomous neurodegeneration in Parkinson’s disease. *Stem Cell Rep.* 12 213–229. 10.1016/j.stemcr.2018.12.011 30639209 PMC6372974

[B26] Eidsvaag V. A. Enger R. Hansson H. A. Eide P. K. Nagelhus E. A. (2017). Human and mouse cortical astrocytes differ in aquaporin-4 polarization toward microvessels. *Glia* 65 964–973. 10.1002/glia.23138 28317216 PMC5413834

[B27] Eng L. F. Vanderhaeghen J. J. Bignami A. Gerstl B. (1971). An acidic protein isolated from fibrous astrocytes. *Brain Res.* 28 351–354. 5113526 10.1016/0006-8993(71)90668-8

[B28] Falcone C. Martínez-Cerdeño V. (2023). Astrocyte evolution and human specificity. *Neural Regen. Res.* 18:131. 10.4103/1673-5374.340405 35799529 PMC9241407

[B29] Falcone C. Penna E. Hong T. Tarantal A. F. Hof P. R. Hopkins W. D. (2021). Cortical interlaminar astrocytes are generated prenatally, mature postnatally, and express unique markers in human and nonhuman primates. *Cereb. Cortex* 31 379–395. 10.1093/cercor/bhaa231 32930323 PMC7947181

[B30] Falcone C. Wolf-Ochoa M. Amina S. Hong T. Vakilzadeh G. Hopkins W. D. (2019). Cortical interlaminar astrocytes across the therian mammal radiation. *J. Comp. Neurol.* 527 1654–1674. 10.1002/cne.24605 30552685 PMC6465161

[B31] Farhy-Tselnicker I. Allen N. J. (2018). Astrocytes, neurons, synapses: A tripartite view on cortical circuit development. *Neural Dev.* 13:7. 10.1186/s13064-018-0104-y 29712572 PMC5928581

[B32] Gao P. Sultan K. T. Zhang X. J. Shi S. H. (2013). Lineage-dependent circuit assembly in the neocortex. *Development* 140 2645–2655. 10.1242/dev.087668 23757410 PMC3678337

[B33] Ge W. P. Miyawaki A. Gage F. H. Jan Y. N. Jan L. Y. (2012). Local generation of glia is a major astrocyte source in postnatal cortex. *Nature* 484 376–380. 10.1038/nature10959 22456708 PMC3777276

[B34] Giaume C. Koulakoff A. Roux L. Holcman D. Rouach N. (2010). Astroglial networks: A step further in neuroglial and gliovascular interactions. *Nat. Rev. Neurosci.* 11 87–99. 10.1038/nrn2757 20087359

[B35] Gressens P. Richelme C. Kadhim H. J. Gadisseux J. F. Evrard P. (1992). The germinative zone produces the most cortical astrocytes after neuronal migration in the developing mammalian brain. *Biol. Neonate* 61 4–24. 10.1159/000243526 1373658

[B36] Guo Q. Scheller A. Huang W. (2021). Progenies of NG2 glia: What do we learn from transgenic mouse models? *Neural Regen. Res.* 16 43–48. 10.4103/1673-5374.286950 32788446 PMC7818854

[B37] Han X. Chen M. Wang F. Windrem M. Wang S. Shanz S. (2013). Forebrain engraftment by human glial progenitor cells enhances synaptic plasticity and learning in adult mice. *Cell Stem Cell* 12 342–353. 10.1016/j.stem.2012.12.015 23472873 PMC3700554

[B38] Hansen D. V. Lui J. H. Parker P. R. Kriegstein A. R. (2010). Neurogenic radial glia in the outer subventricular zone of human neocortex. *Nature* 464 554–561. 10.1038/nature08845 20154730

[B39] Holst C. B. Brøchner C. B. Vitting-Seerup K. Møllgård K. (2019). Astrogliogenesis in human fetal brain: Complex spatiotemporal immunoreactivity patterns of GFAP, S100, AQP4 and YKL-40. *J. Anat.* 235 590–615. 10.1111/joa.12948 30901080 PMC6704246

[B40] Iadecola C. (2017). The neurovascular unit coming of age: A journey through neurovascular coupling in health and disease. *Neuron* 96 17–42. 10.1016/j.neuron.2017.07.030 28957666 PMC5657612

[B41] Iliff J. J. Nedergaard M. (2013). Is there a cerebral lymphatic system? *Stroke* 44(6 Suppl 1) S93–S95. 10.1161/strokeaha.112.678698 23709744 PMC3699410

[B42] Jacobsen C. T. Miller R. H. (2003). Control of astrocyte migration in the developing cerebral cortex. *Dev. Neurosci.* 25 207–216. 10.1159/000072269 12966218

[B43] Jessen N. A. Munk A. S. F. Lundgaard I. Nedergaard M. (2015). The glymphatic system: A beginner’s guide. *Neurochem. Res.* 40 2583–2599. 10.1007/s11064-015-1581-6 25947369 PMC4636982

[B44] Khakh B. S. Sofroniew M. V. (2015). Diversity of astrocyte functions and phenotypes in neural circuits. *Nat. Neurosci.* 18 942–952. 10.1038/nn.4043 26108722 PMC5258184

[B45] Kiyoshi C. M. Du Y. Zhong S. Wang W. Taylor A. T. Xiong B. (2018). Syncytial isopotentiality: A system-wide electrical feature of astrocytic networks in the brain. *Glia* 66 2756–2769. 10.1002/glia.23525 30277621 PMC8818325

[B46] Lamonica B. E. Lui J. H. Hansen D. v Kriegstein A. R. (2013). Mitotic spindle orientation predicts outer radial glial cell generation in human neocortex. *Nat. Commun.* 4:1665. 10.1038/ncomms2647 23575669 PMC3625970

[B47] Li J. Pan L. Pembroke W. G. Rexach J. E. Godoy M. I. Condro M. C. (2021). Conservation and divergence of vulnerability and responses to stressors between human and mouse astrocytes. *Nat. Commun.* 12:3958. 10.1038/s41467-021-24232-3 34172753 PMC8233314

[B48] Li X. Liu G. Yang L. Li Z. Zhang Z. Xu Z. (2021). Decoding cortical glial cell development. *Neurosci. Bull.* 37 440–460. 10.1007/s12264-021-00640-9 33606177 PMC8055813

[B49] Lin Y. T. Seo J. Gao F. Feldman H. M. Wen H. L. Penney J. (2018). APOE4 causes widespread molecular and cellular alterations associated with Alzheimer’s disease phenotypes in human iPSC-derived brain cell types. *Neuron* 98 1141.e–1154.e. 10.1016/j.neuron.2018.05.008 29861287 PMC6023751

[B50] Ma B. Buckalew R. Du Y. Kiyoshi C. M. Alford C. C. Wang W. (2016). Gap junction coupling confers isopotentiality on astrocyte syncytium. *Glia* 64 214–226. 10.1002/glia.22924 26435164 PMC4595908

[B51] Macvicar B. A. Newman E. A. (2015). Astrocyte regulation of blood flow in the brain. *Cold Spring Harb. Perspect. Biol.* 7 1–15. 10.1101/cshperspect.a020388 25818565 PMC4448617

[B52] Malik S. Vinukonda G. Vose L. R. Diamond D. Bhimavarapu B. B. R. Hu F. (2013). Neurogenesis continues in the third trimester of pregnancy and is suppressed by premature birth. *J. Neurosci.* 33 411–423. 10.1523/JNEUROSCI.4445-12.2013 23303921 PMC3711635

[B53] Maoz B. M. Herland A. Fitzgerald E. A. Grevesse T. Vidoudez C. Pacheco A. R. (2018). A linked organ-on-chip model of the human neurovascular unit reveals the metabolic coupling of endothelial and neuronal cells. *Nat. Biotechnol.* 36 865–877. 10.1038/nbt.4226 30125269 PMC9254231

[B54] Miyata T. Kawaguchi A. Okano H. Ogawa M. (2001). Asymmetric inheritance of radial glial fibers by cortical neurons. *Neuron* 31 727–741. 10.1016/s0896-6273(01)00420-2 11567613

[B55] Molnár Z. Clowry G. J. Šestan N. Alzu’bi A. Bakken T. Hevner R. F. (2019). New insights into the development of the human cerebral cortex. *J. Anat.* 235 432–451. 10.1111/joa.13055 31373394 PMC6704245

[B56] Molofsky A. V. Deneen B. (2015). Astrocyte development: A guide for the perplexed. *Glia* 63 1320–1329. 10.1002/glia.22836 25963996

[B57] Mukhtar T. Taylor V. (2018). Untangling cortical complexity during development. *J. Exp. Neurosci.* 12:1179069518759332. 10.1177/1179069518759332 29551911 PMC5846925

[B58] Nadarajah B. Brunstrom J. E. Grutzendler J. Wong R. O. L. Pearlman A. L. (2001). Two modes of radial migration in early development of the cerebral cortex. *Nat. Neurosci.* 4 143–150. 10.1038/83967 11175874

[B59] Nagelhus E. A. Ottersen O. P. (2013). Physiological roles of aquaporin-4 in brain. *Physiol. Rev.* 93 1543–1562. 10.1152/physrev.00011.2013 24137016 PMC3858210

[B60] Nagy J. I. Patel D. Ochalski P. A. Y. Stelmack G. L. (1999). Connexin30 in rodent, cat and human brain: Selective expression in gray matter astrocytes, co-localization with connexin43 at gap junctions and late developmental appearance. *Neuroscience* 88 447–468. 10.1016/s0306-4522(98)00191-2 10197766

[B61] Namba T. Huttner W. B. (2017). Neural progenitor cells and their role in the development and evolutionary expansion of the neocortex. *Wiley Interdiscip. Rev. Dev. Biol.* 6. 10.1002/wdev.256 27865053

[B62] Nedergaard M. Ransom B. Goldman S. A. (2003). New roles for astrocytes: Redefining the functional architecture of the brain. *Trends Neurosci.* 26 523–530. 10.1016/j.tins.2003.08.008 14522144

[B63] Nishiyama A. Boshans L. Goncalves C. M. Wegrzyn J. Patel K. D. (2016). Lineage, fate, and fate potential of NG2-glia. *Brain Res.* 1638 116–128. 10.1016/J.BRAINRES.2015.08.013 26301825 PMC4761528

[B64] Noctor S. C. Martinez-Cerdeño V. Ivic L. Kriegstein A. R. (2004). Cortical neurons arise in symmetric and asymmetric division zones and migrate through specific phases. *Nat. Neurosci.* 7 136–144. 10.1038/nn1172 14703572

[B65] Nowakowski T. J. Pollen A. A. Sandoval-Espinosa C. Kriegstein A. R. (2016). Transformation of the radial glia scaffold demarcates two stages of human cerebral cortex development. *Neuron* 91 1219–1227. 10.1016/j.neuron.2016.09.005 27657449 PMC5087333

[B66] Oberheim N. A. Takano T. Han X. He W. Lin J. H. C. Wang F. (2009). Uniquely hominid features of adult human astrocytes. *J. Neurosci.* 29 3276–3287. 10.1523/JNEUROSCI.4707-08.2009 19279265 PMC2819812

[B67] Ortega J. A. Memi F. Radonjic N. Filipovic R. Bagasrawala I. Zecevic N. (2018). The subventricular zone: A key player in human neocortical development. *Neuroscientist* 24 156–170. 10.1177/1073858417691009 29254416

[B68] Osipovitch M. Asenjo Martinez A. Mariani J. N. Cornwell A. Dhaliwal S. Zou L. (2019). Human ESC-derived chimeric mouse models of Huntington’s disease reveal cell-intrinsic defects in glial progenitor cell differentiation. *Cell Stem Cell* 24 107–122.e7. 10.1016/j.stem.2018.11.010 30554964 PMC6700734

[B69] Ota Y. Zanetti A. T. Hallock R. M. (2013). The role of astrocytes in the regulation of synaptic plasticity and memory formation. *Neural Plast.* 2013:185463. 10.1155/2013/185463 24369508 PMC3867861

[B70] Parpura V. Basarsky T. A. Liu F. Jeftinija K. Jeftinija S. Haydon P. G. (1994). Glutamate-mediated astrocyte-neuron signalling. *Nature* 369 744–747. 10.1038/369744a0 7911978

[B71] Perea G. Navarrete M. Araque A. (2009). Tripartite synapses: Astrocytes process and control synaptic information. *Trends Neurosci.* 32 421–431. 10.1016/j.tins.2009.05.001 19615761

[B72] Qi X. R. Verwer R. W. H. Bao A. M. Balesar R. A. Luchetti S. Zhou J. N. (2019). Human brain slice culture: A useful tool to study brain disorders and potential therapeutic compounds. *Neurosci. Bull.* 35 244–252. 10.1007/s12264-018-0328-1 30604279 PMC6426918

[B73] Rakic P. (1988). Specification of cerebral cortical areas. *Science* 241 170–176. 10.1126/science.3291116 3291116

[B74] Rakic P. (2009). Evolution of the neocortex: A perspective from developmental biology. *Nat. Rev. Neurosci.* 10 724–735. 10.1038/nrn2719 19763105 PMC2913577

[B75] Ramón y Cajal S. (1913). Un nuevo proceder para la impregnación de la neuroglía. *Bol. Soc. Esp. Bio.* 2 104–108.

[B76] Rash B. G. Duque A. Morozov Y. M. Arellano J. I. Micali N. Rakic P. (2019). Gliogenesis in the outer subventricular zone promotes enlargement and gyrification of the primate cerebrum. *Proc. Natl. Acad. Sci. U.S.A.* 116 7089–7094. 10.1073/pnas.1822169116 30894491 PMC6452694

[B77] Retzius G. (1894). Die neuroglia des gehirns beim menschen und bei säugethieren. *Biol. Untersuchungen* 6 1–28.

[B78] Roessmann U. Gambetti P. (1986). Astrocytes in the developing human brain. An immunohistochemical study. *Acta Neuropathol.* 70 308–313. 10.1007/BF00686089 3766128

[B79] Rouach N. Avignone E. Même W. Koulakoff A. Venance L. Blomstrand F. (2002). Gap junctions and connexin expression in the normal and pathological central nervous system. *Biol. Cell* 94 457–475. 10.1016/S0248-4900(02)00016-3 12566220

[B80] Schwarz N. Hedrich U. Schwarz H. P A H. Dammeier N. Auffenberg E. (2017). Human Cerebrospinal fluid promotes long-term neuronal viability and network function in human neocortical organotypic brain slice cultures. *Sci. Rep.* 7:12249. 10.1038/s41598-017-12527-9 28947761 PMC5613008

[B81] Simard M. Nedergaard M. (2004). The neurobiology of glia in the context of water and ion homeostasis. *Neuroscience* 129 877–896. 10.1016/j.neuroscience.2004.09.053 15561405

[B82] Siracusa R. Fusco R. Cuzzocrea S. (2019). Astrocytes: Role and functions in brain pathologies. *Front. Pharmacol.* 10:1114. 10.3389/fphar.2019.01114 31611796 PMC6777416

[B83] Sloan S. A. Darmanis S. Huber N. Khan T. A. Birey F. Caneda C. (2017). Human astrocyte maturation captured in 3D cerebral cortical spheroids derived from pluripotent stem cells. *Neuron* 95 779–790.e6. 10.1016/j.neuron.2017.07.035 28817799 PMC5890820

[B84] Sonnewald U. Westergaard N. Schousboe A. (1997). Glutamate transport and metabolism in astrocytes. *Glia* 21 56–63. 10.1002/(SICI)1098-1136(199709)21:1<56::AID-GLIA6>3.0.CO;2-# 9298847

[B85] Sosunov A. A. Wu X. Tsankova N. M. Guilfoyle E. McKhann G. M. II Goldman J. E. (2014). Phenotypic heterogeneity and plasticity of isocortical and hippocampal astrocytes in the human brain. *J. Neurosci.* 34, 2285–2298. 10.1523/JNEUROSCI.4037-13.2014 24501367 PMC3913872

[B86] Tabata H. (2015). Diverse subtypes of astrocytes and their development during corticogenesis. *Front. Neurosci.* 9:114. 10.3389/fnins.2015.00114 25904839 PMC4387540

[B87] Tchieu J. Calder E. L. Guttikonda S. R. Gutzwiller E. M. Aromolaran K. A. Steinbeck J. A. (2019). NFIA is a gliogenic switch enabling rapid derivation of functional human astrocytes from pluripotent stem cells. *Nat. Biotechnol.* 37 267–275. 10.1038/s41587-019-0035-0 30804533 PMC6591152

[B88] Varcianna A. Myszczynska M. A. Castelli L. M. O’Neill B. Kim Y. Talbot J. (2019). Micro-RNAs secreted through astrocyte-derived extracellular vesicles cause neuronal network degeneration in C9orf72 ALS. *EBiomedicine* 40 626–635. 10.1016/j.ebiom.2018.11.067 30711519 PMC6413467

[B89] Vasile F. Dossi E. Rouach N. (2017). Human astrocytes: Structure and functions in the healthy brain. *Brain Struct. Funct.* 222 2017–2029. 10.1007/s00429-017-1383-5 28280934 PMC5504258

[B90] Vatine G. D. Barrile R. Workman M. J. Sances S. Barriga B. K. Rahnama M. (2019). Human iPSC-derived blood-brain barrier chips enable disease modeling and personalized medicine applications. *Cell Stem Cell* 24 995–1005.e6. 10.1016/j.stem.2019.05.011 31173718

[B91] Verkhratsky A. Kettenmann H. (1996). Calcium signalling in glial cells. *Trends Neurosci.* 19 346–352. 10.1016/0166-2236(96)10048-5 8843604

[B92] Verkhratsky A. Nedergaard M. (2018). Physiology of astroglia. *Physiol. Rev.* 98, 239–389. 10.1152/physrev.00042.2016 29351512 PMC6050349

[B93] Verkhratsky A. Parpura V. Li B. Scuderi C. (2021). “Astrocytes: The housekeepers and guardians of the CNS,” in *Astrocytes in Psychiatric Disorders Advances in Neurobiology*, eds Li B. Parpura V. Verkhratsky A. Scuderi C. (Cham: Springer), 21–53. 10.1007/978-3-030-77375-5_2 PMC9004589 34888829

[B94] Virchow R. (1856). “Ueber das granulirte Ansehen der Wandungen der Gehirnventrikel,” in *Gesammelte Abhandlungen zur wissenschaftlichen Medicin*, ed. Virchow R. (Frankfurt: Meidinger Sohn & Comp), 885–891.

[B95] Virchow R. (1858). *Cellular Pathology: As Based Upon Physiological and Pathological Histology*, 1st Edn. Berlin: Hirschwald. 10.1111/j.1753-4887.1989.tb02747.x 2649802

[B96] Yang L. Li Z. Liu G. Li X. Yang Z. (2022). Developmental origins of human cortical oligodendrocytes and astrocytes. *Neurosci. Bull.* 38 47–68. 10.1007/s12264-021 34374948 PMC8783027

[B97] Zhang Y. Sloan S. A. Clarke L. E. Caneda C. Plaza C. A. Blumenthal P. D. (2016). Purification and characterization of progenitor and mature human astrocytes reveals transcriptional and functional differences with mouse. *Neuron* 89 37–53. 10.1016/j.neuron.2015.11.013 26687838 PMC4707064

[B98] Zhong S. Kiyoshi C. M. Du Y. Wang W. Luo Y. Wu X. (2023). Genesis of a functional astrocyte syncytium in the developing mouse hippocampus. *Glia* 71 1081–1098. 10.1002/glia.24327 36598109 PMC10777263

